# Supplementary material for: A Critical View of the Application of the APEX Software (Aqueous Photochemistry of Environmentally-Occurring Xenobiotics) to Predict Photoreaction Kinetics in Surface Freshwaters
Source: Molecules. 2019 Dec 18;25(1):9. doi: 10.3390/molecules25010009 (PMC7017383; doi:10.3390/molecules25010009)
Supplement: Supplementary file 1 [file molecules-25-00009-s001.zip › 3.2.4_gcc-4.4.0.zip!/3.2.4_gcc-4.4.0/3.2.4_gcc-4.4.0/doc/octave/html/interpreter/Conversion-of-Numerical-Data-to-Strings.html]

Conversion of Numerical Data to Strings - Untitled


Previous: Concatenating Strings,
Up: Creating Strings


---

#### 5.3.2 Conversion of Numerical Data to Strings

Apart from the string concatenation functions (see Concatenating Strings)
which cast numerical data to the corresponding ASCII characters, there are
several functions that format numerical data as strings. `mat2str` and
`num2str` convert real or complex matrices, while `int2str` converts
integer matrices. `int2str` takes the real part of complex values and
round fractional values to integer. A more flexible way to format numerical
data as strings is the `sprintf` function (see Formatted Output,
doc-sprintf).

— Function File: s = **mat2str** (x, n)  
— Function File: s = **mat2str** (..., 'class')  
> Format real/complex numerical matrices as strings. This function
> returns values that are suitable for the use of the `eval`
> function.
>
> The precision of the values is given by n. If n is a
> scalar then both real and imaginary parts of the matrix are printed
> to the same precision. Otherwise n `(1)` defines the
> precision of the real part and n `(2)` defines the
> precision of the imaginary part. The default for n is 17.
>
> If the argument 'class' is given, then the class of x is
> included in the string in such a way that the eval will result in the
> construction of a matrix of the same class.
>
> ```
>           mat2str ([ -1/3 + i/7; 1/3 - i/7 ], [4 2])
>                => "[-0.3333+0.14i;0.3333-0.14i]"
>           
>           mat2str ([ -1/3 +i/7; 1/3 -i/7 ], [4 2])
>                => "[-0.3333+0i,0+0.14i;0.3333+0i,-0-0.14i]"
>           
>           mat2str (int16([1 -1]), 'class')
>                => "int16([1,-1])"
> ```
>
>
>
>
> **See also:** sprintf, num2str, int2str.

— Function File: **num2str** (x)  
— Function File: **num2str** (x, precision)  
— Function File: **num2str** (x, format)  
> Convert a number (or array) to a string (or a character array). The
> optional second argument may either give the number of significant
> digits (precision) to be used in the output or a format
> template string (format) as in `sprintf` (see Formatted Output). `num2str` can also handle complex numbers. For
> example:
>
> ```
>           num2str (123.456)
>                => "123.46"
>           
>           num2str (123.456, 4)
>                => "123.5"
>           
>           s = num2str ([1, 1.34; 3, 3.56], "%5.1f")
>                => s =
>                   1.0  1.3
>                   3.0  3.6
>           whos s
>                =>
>                 Attr Name        Size                     Bytes  Class
>                 ==== ====        ====                     =====  =====
>                      s           2x8                         16  char
>           
>           num2str (1.234 + 27.3i)
>                => "1.234+27.3i"
> ```
>
> The `num2str` function is not very flexible. For better control
> over the results, use `sprintf` (see Formatted Output).
> Note that for complex x, the format string may only contain one
> output conversion specification and nothing else. Otherwise, you
> will get unpredictable results.
>
>
>
> **See also:** sprintf, int2str, mat2str.

— Function File: **int2str** (n)  
> Convert an integer (or array of integers) to a string (or a character
> array).
>
> ```
>           int2str (123)
>                => "123"
>           
>           s = int2str ([1, 2, 3; 4, 5, 6])
>                => s =
>                   1  2  3
>                   4  5  6
>           
>           whos s
>                => s =
>                 Attr Name        Size                     Bytes  Class
>                 ==== ====        ====                     =====  =====
>                      s           2x7                         14  char
> ```
>
> This function is not very flexible. For better control over the
> results, use `sprintf` (see Formatted Output).
>
>
>
> **See also:** sprintf, num2str, mat2str.
